# Evaluation of Trunk Muscle Mass in Older Adults Using Bioelectrical Impedance Analysis: A Scoping Review

**DOI:** 10.31662/jmaj.2025-0083

**Published:** 2025-08-08

**Authors:** Ryo Shiraishi, Masatoshi Nakamura, Shunji Araki, Masanobu Yokochi

**Affiliations:** 1 PREVENT Inc, Higashi, Japan; 2 Department of Rehabilitation Medicine, Aichi Medical University, Nagakute, Japan; 3 Faculty of Rehabilitation Sciences, Nishi Kyushu University, Kanzaki, Japan; 4 Clinical Education and Research Center, Chuzan Hospital, Okinawa, Japan; 5 Department of Rehabilitation, Takeda General Hospital, Aizuwakamatsu, Japan

**Keywords:** scoping review, bioelectrical impedance analysis, muscle mass, muscle composition, muscle architecture

## Abstract

**Background:**

Trunk muscle mass decreases with age; this decrease is reported to be related to physical function and activities of daily living. Bioelectrical impedance analysis (BIA) may be used to effectively evaluate trunk muscle mass as an alternative to conventional measurement methods such as computed tomography and magnetic resonance imaging. This scoping review aimed to comprehensively examine existing knowledge on types and frequency of BIA devices and the relationship between trunk muscle mass and clinical outcomes.

**Methods:**

We systematically reviewed studies involving older adults aged ≥65 years. We searched PubMed, Web of Science, and Scopus databases; observational studies (cohort, case-control, and cross-sectional) published between January 2000 and October 2023 were reviewed. Three independent reviewers summarized the BIA device model, BIA frequency, study design, study objectives, and outcomes.

**Results:**

In total, 7 of 744 identified studies were reviewed. Most participants included in this review were community-dwelling older adults and older patients with stroke. The main research design was a prospective or retrospective cohort study. In addition, a multi-frequency model was used for the BIA device. Common clinical outcome indicators were physical function (swallowing) and activities of daily living.

**Conclusions:**

The findings of this review suggest that BIA is an effective method for evaluating trunk muscle mass in older adults. However, the causal relationship between trunk muscle mass and clinical outcomes is unclear, necessitating the accumulation of more data from additional clinical studies.

## Introduction

Diseases characterized by progressive loss of muscle mass in older adults are an important challenge ^(1)^. Particularly, among skeletal muscles, the trunk muscles show a decrease in muscle mass with age ^(2)^. The loss of trunk muscle mass in older adults is associated with decreased balance and increased falls ^(3), (4)^, and reduced trunk muscle mass is associated with decreased walking ability ^(5)^. Furthermore, a decrease in trunk muscle mass in older adults is associated with changes in posture and a decrease in activities of daily living (ADLs) ^(6), (7)^. Therefore, the amount of muscle mass in the trunk is considered to be important in older adults.

Traditionally, computed tomography (CT), magnetic resonance imaging (MRI), and dual-energy X-ray absorption (DXA) have been the gold standards for evaluating trunk muscle mass ^(8), (9), (10)^. These methods generally trace muscle cross-sectional area, often using the thoracic or lumbar spine as the measurement site ^(11), (12), (13), (14)^. However, their clinical use is limited owing to high cost, time consumption, and patient burden. Bioelectrical impedance analysis (BIA) offers a useful method for measuring muscle mass, including appendicular muscle mass ^(15), (16), (17)^. BIA measures muscle mass by applying weak electricity to the body and using a calculation formula in the measurement device based on electrical resistance ^(18)^. In addition, as the measuring device is portable, BIA can be easily used at the bedside and measures muscle mass with minimal invasiveness ^(19), (20)^. These results indicate that BIA is a useful alternative to MRI and DXA for evaluating appendicular muscle mass.

Body composition in older adults has been investigated previously. Recently, BIA assessment has reported trunk muscle mass in older adults. However, few reviews have focused on the type of BIA device or frequency used to evaluate trunk muscle mass. Therefore, the evaluation of trunk muscle mass using BIA is still in its nascent stages, and there is a pressing need for its validation. To the best of our knowledge, there have been no reviews that have investigated both the trunk muscle mass using BIA assessment in this population and the relationship between trunk muscle mass and functional outcomes such as physical function and ADL. Therefore, it is important to review existing research on trunk muscle mass in older adults and identify knowledge gaps. Accordingly, we aimed to comprehensively survey and analyze existing findings on types and frequencies of BIA devices and the relationship between trunk muscle mass and clinical outcomes to guide future research.

## Materials and Methods

This study used a scoping review method to comprehensively search for and address existing studies related to the research question. The review was conducted using the Joanna Briggs Institute framework ^(21)^, which broadly involves creating a scoping review protocol and summarizing the literature. The development of a scoping review protocol includes identifying the research question and selection criteria, search methods, literature selection, data extraction, literature analysis, and presentation of results. This process is followed by a summary of the literature. Scoping reviews were reported according to the Preferred Reporting Items for Systematic Review and Meta-Analyses (PRISMA) Extension for Scoping Reviews.

### Eligibility criteria

The framework for the scoping review included “Participants,” “Concept,” and “Context.” Participants refer to the characteristics and age of participants, who were defined as older adults in this study, regardless of whether they had a disease. In this study, older adults were defined as those aged ≥65 years, in accordance with the World Health Organization (WHO) definition. Concept refers to the main concepts considered in a scoping review, and include “evaluation methods,” “effectiveness indicators to evaluate measurements,” and “study design.” The concept of this study was to investigate studies that evaluated trunk muscle mass using BIA. Context typically limits the scope of the search to specific countries, regions, or facilities, depending on the research question; however, it was not used in this study. Only observational studies (cohort, case-control, and cross-sectional studies) were included, excluding intervention studies and review articles.

### Search strategy

In this study, a comprehensive search was conducted using PubMed, Web of Science, and Scopus databases. In addition, we manually searched the database to identify relevant papers that were not collected in the initial database search, yet were similar or cited in other studies. The primary search terms were “trunk muscle mass” and “bioelectrical impedance analysis”. The database search period included studies published between January 2000 and October 2023, with the last search conducted on October 31, 2023. This study included reports that were written and published in English.

### Selection process

The searched studies were uploaded to Rayyan ^(22)^ (Qatar Computing Research Institute, Ar Rayyan, Qatar), and duplicate studies were removed. Duplicate studies were excluded from the search results using each database. Subsequently, three reviewers independently evaluated the papers based on their titles and abstracts, adhering to the inclusion criteria. In addition, the full text of the applicable articles was evaluated, and detailed reasons for article exclusion were recorded. This process was documented in a flow chart. Any disagreements between the reviewers at each stage of the study selection process were reported to a fourth reviewer and resolved through discussion.

### Data extraction

Data extraction was performed using data sheets prepared by three independent reviewers. Data extracted included the author of the article, year of publication, country, disease of interest, age, sex, BMI, sample size, BIA device model, BIA frequency, sensitivity/specificity, study design, study objectives, and outcomes.

## Results

### Research selection

**Figure 1.**
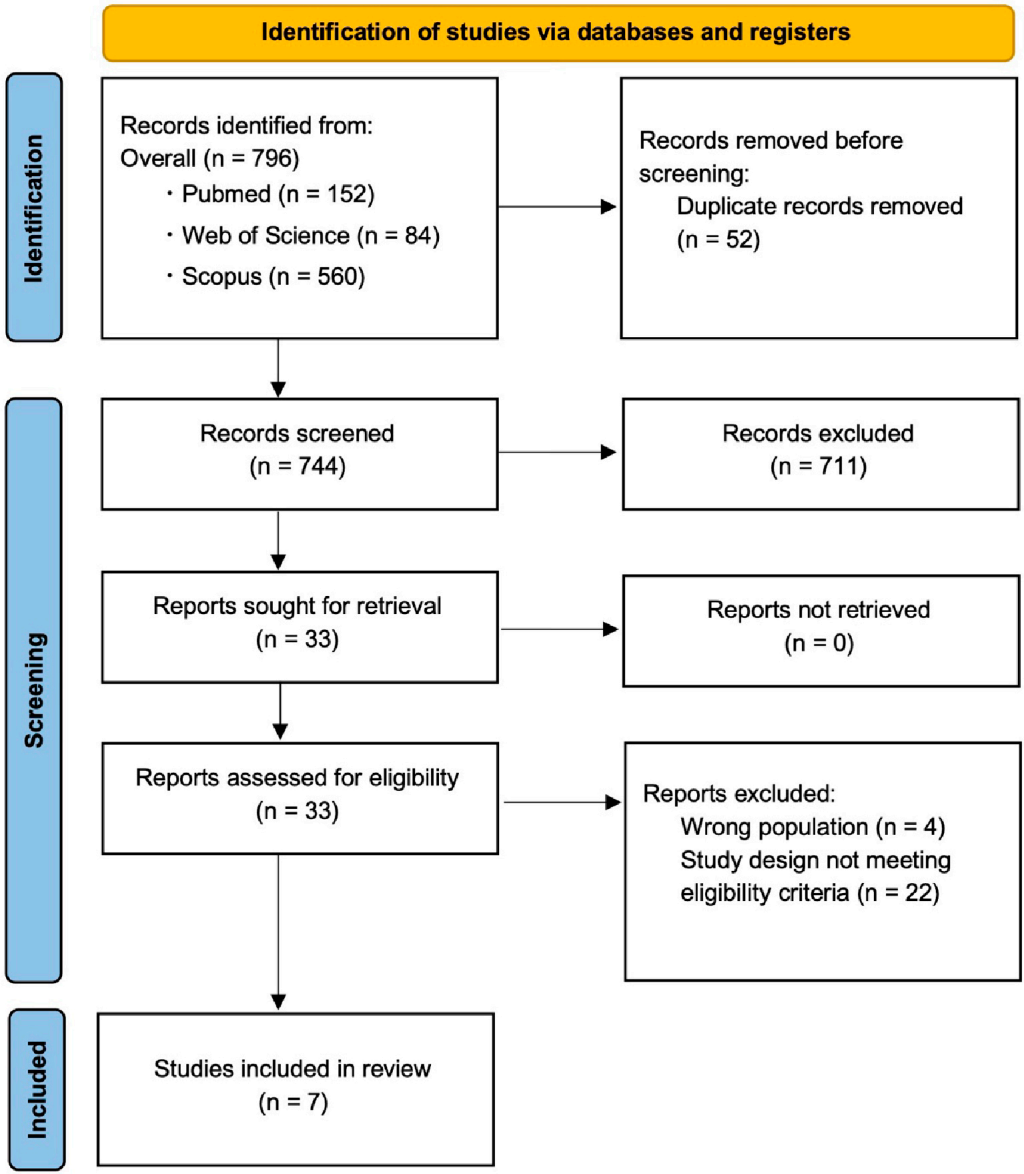
A PRISMA flowchart of the selection criteria. PRISMA: Preferred Reporting Items for Systematic Review and Meta-Analyses.

A PRISMA flowchart of the selection criteria for the scoping review of this study is shown in Figure 1 (Search date: October 31, 2023). After removing duplicate papers, a total of 744 papers were screened first by title and abstract. The 33 original articles that remained from the primary screening were obtained and screened in detail by the full text (secondary screening). Out of 33 papers, seven papers were selected for data extraction based on the selection criteria.

### Overview of selected studies

Table 1 provides a summary of the included studies, including the year of publication, country, study population, and age of the participants. The seven selected papers were reported from 2018 to 2023. All seven studies were conducted in Japan.

**Table 1.** Research Overview and Main Research Findings.

Authors (year)	Country	Study population	Study design	Sample size	Age (mean)	Sex (n; %)	BMI (kg/m^2^)	BIA device model	BIA frequencies	Sensitivity/specificity	Main objectives	Main outcomes
Yamaguchi et al. ^(29)^, 2023	Japan	Community-dwelling older adults	Cross-sectional study	141	71.6 ± 4.7 (men: 72.5 ± 5.2; women: 71.1 ± 4.5)	men: 46; women: 95	23.1 ± 2.7 (men: 23.0 ± 3.0; women: 23.1 ± 2.6)	In Body S10 (Inbody, Tokyo, Japan)	multifrequency alternating current of 1, 5, 50, 250, 500, and 1000 kHz	N/A	To clarify the relationship between TMI and the characteristics of swallowing-related muscles.	For both men and women, the CSA of the geniohyoid muscle was significantly positively correlated with TMI (r = 0.50 [men], r = 0.21, p < 0.05 [women]). TMI was significantly and positively associated with the swallowing-related muscle mass.
Ide et al. ^(6)^, 2023	Japan	Older adults	Cross-sectional study	281	75.4 ± 6.7 (men: 75.9 ± 6.8; women: 75.1 ± 6.7)	men: 100; women: 181	22.7 ± 3.0 (men: 23.3 ± 2.8; women: 22.3 ± 3.1)	MC-780A (Tanita, Tokyo, Japan)	multifrequency alternating current of 5, 50, 250 kHz	N/A	To investigate the relationship between TMM and whole-body sagittal alignment.	TMM decreased with age. In women, TMM/BMI decreased, but not in men. TMM and TMM/BMI were associated with trunk forward inclination (men: r = 0.275, women: r = 0.300, p < 0.001) and knee flexion (men: r = 0.305, women: r = 0.362). In addition, TMM/BMI was significantly correlated with the Oswestry Disability Index (men: r = 0.256, women: r = 0.214).
Sato et al. ^(25)^, 2022	Japan	Patients with acute stroke	Retrospective cohort study	212	72.7 [13.4]	men: 143; women: 69	23.6 ± 3.8	In Body S10 (Inbody, Tokyo, Japan)	multifrequency alternating current of 1, 5, 50, 250, 500, and 1000 kHz	N/A	To determine whether TMI is associated with functional prognosis and to determine whether each motor subitem was related to TMI.	TMI showed a positive correlation with the FIM-motor score at the time of discharge (β = 0.240). FIM-self-care (β = 0.351) and locomotion (β = 0.331) were positively associated with TMI.
Sato et al. ^(28)^, 2022	Japan	Patients with acute stroke	Retrospective cohort study	231	72.2 [13.3]	men: 151; women: 80	23.6 ± 3.7	In Body S10 (Inbody, Tokyo, Japan)	multifrequency alternating current of 1, 5, 50, 250, 500, and 1000 kHz	N/A	To determine the effect of TMI on improving swallowing function in patients with acute stroke.	TMI was related to FIM-eating score (β = 0.330).
Salimi et al. ^(13)^, 2021	Japan	Community-dwelling older adults	Prospective cohort study	380	73.4 [5.3] (men: 73.7 [5.2]; women: 73.3 [5.5])	men: 152; women: 228	23.0 ± 3.3 (men: 23.4 ± 2.8; women: 22.7 ± 3.6)	MC-780A (Tanita, Tokyo, Japan)	multifrequency alternating current of 5, 50, 250 kHz	N/A	To verify whether TMM measured using BIA correlates with muscle cross-sectional area of paravertebral muscles measured using MRI.	A strong correlation (r = 0.807) was shown between TMM measured using BIA and the cross-sectional area of the paravertebral muscles (excluding intramuscular fat) measured using MRI.
Hori et al. ^(27)^, 2019	Japan	Patients with spinal diseases	Cross-sectional study	1738	70.2 ± 11.0	men: 781; women: 957	23.3 ± 3.9	MC-780A and MC-980A (Tanita, Tokyo, Japan)	multifrequency alternating current of 1, 5, 50, 250, 500, and 1000 kHz	N/A	To clarify the relationship between TMM and low back pain, spinal sagittal balance, and QOL, and to elucidate the significance of TMM in patients with spinal diseases.	TMM as assessed by BIA was significantly associated with ODI, low back pain VAS score, SVA, and EQ5D.
Yoshimi et al. ^(26)^, 2018	Japan	Community-dwelling older adults	Cross-sectional study	118	men: 72.81 (69.00-77.00); women: 69.57 (66.00-73.00)	men: 37 women: 81	men: 23.02 ± 2.65; women: 22.68 ± 3.15	In Body S10 (Inbody, Tokyo, Japan)	multifrequency alternating current of 1, 5, 50, 250, 500, and 1000 kHz	N/A	To clarify the relationship between the strength of swallowing muscles and TMM in healthy older individuals.	A correlation was found between handgrip strength and geniohyoid muscle cross-sectional area in both men and women (men: r = 0.508, women: r = 0.279). The geniohyoid muscle cross-sectional area was correlated with TMI (r = 0.290). TMI was one of the factors related to JOF (β = 0.299) and TP (β = 0.195).

BIA: bioelectrical impedance analysis; BMI: body mass index; TMI: trunk muscle mass index; CSA: cross-sectional area; TMM: trunk muscle mass; FIM: functional independence measure; MRI: magnetic resonance imaging; QOL: quality of life; ODI: Oswestry Disability Index; VAS: visual analog scale; SVA: sagittal vertical axis; EQ5D: EuroQol 5 Dimension; JOF: jaw-opening force; TP: tongue pressure.

### Study participants

The study participants comprised older adults living in the community and patients with stroke in the acute phase of the disease (Table 1).

### Study design

The study designs included prospective cohort studies (10%), retrospective cohort studies (30%), and cross-sectional studies (60%), as detailed in Table 1.

### BIA model and frequency

The BIA model used to measure trunk muscle mass was an 8-pole model, employing frequencies of 1, 5, 50, 250, 500, and 1000 kHz (Table 1).

### Study objectives and outcomes

This study report investigated the association between trunk muscle mass and clinical outcomes such as ADLs, physical function (swallowing), and postural alignment (Table 1).

## Discussion

This scoping review systematically searched and summarized studies on trunk muscle mass evaluated by BIA in older adults, all conducted in Japan. The review focused on the BIA device model and frequency, study objectives, and study outcomes. Most participants were older adults from the community and older patients with stroke. Despite a small number of studies (seven papers) in the past 5 years, potential for future development in this field exists.

Traditionally, CT, MRI, and DXA have been the gold standards for evaluating trunk muscle mass ^(14), (23), (24)^. However, owing to patient burden, time constraints, and costs, they are rarely used in clinical practice. Hence, BIA was developed as a simpler alternative ^(18)^. Although BIA is primarily used to evaluate skeletal muscles of the extremities, it is also employed for evaluating muscle mass at different sites. A strong correlation exists between trunk muscle cross-sectional area measured using MRI and trunk muscle mass assessed using BIA in older community-dwelling adults ^(13)^. Therefore, BIA-evaluated trunk muscle mass may be a valuable indicator. However, this is the only study that has examined the validity of conventional measurement instruments versus BIA. Further validation studies are essential to confirm the accuracy of trunk muscle mass evaluation using BIA.

All studies included in this review used multi-frequency BIA devices (In Body S10, Inbody, Tokyo, Japan, and MC-780A, Tanita, Tokyo, Japan) ^(6), (13), (25), (26), (27), (28), (29)^. Multi-frequency devices may provide more accurate body composition estimates than single-frequency bioelectrical devices ^(30)^. However, differing opinions exist regarding the interpretation of muscle mass evaluated by BIA, with some concerns raised about potential errors owing to water content ^(31), (32)^. Therefore, the value of muscle mass evaluated may be over-interpreted. In addition, more attention should be paid to the trunk muscle mass over extremity muscle mass owing to its inclusion of internal organs. BIA can measure intracellular water, extracellular water (ECW), and total body water (TBW) in addition to muscle mass ^(15), (33), (34), (35)^. An increase in ECW or the ECW/TBW ratio suggests systemic edema ^(34)^, highlighting the importance of evaluating muscle mass results obtained from the BIA against these indices. Therefore, recognizing that BIA data on trunk muscle mass may exhibit substantial variations based on age, sex, race, and health status. Multi-frequency BIA devices can be used to measure trunk muscle mass; this review specifically investigated two types of measurement devices. However, multi-frequency BIA devices come in various models and protocols. Therefore, it is important to note that the measurement procedure may differ depending on the model of the measurement device and that there is no standardization. In the future, further efforts are needed to standardize measurement devices and unify measurement procedures.

This review only includes research published in Japan. However, BIA is a body composition analysis method that is used not only in Japan but also in many other countries. In addition, the evaluation of muscle mass in body composition is generally based on the extremities. Furthermore, there is consensus on the evaluation of muscle mass using the four limbs, and its validity has been reported. However, there is no consensus on the evaluation of trunk muscle mass using BIA, and this method is not commonly used to evaluate muscle mass. In Japan, Yoshimi et al. ^(26)^ first reported on the evaluation of trunk muscle mass using BIA in 2018. Thereafter, reports from Japan began to appear sporadically. As Japanese participants tend to be ethnically homogeneous, using these reports as a reference for evaluation methods is easier. In the future, it is possible that a consensus will be reached owing to many studies being published in this field.

Trunk muscle mass and its relation to clinical outcomes as evaluated by BIA were described in this review. Most studies included in this review investigated associations with functional outcomes such as physical function and ADLs ^(6), (13), (25), (26), (27), (28), (29)^. Trunk muscles contribute to body stability and postural maintenance and serve as antigravity muscles controlling spinal column and pelvic movement. Therefore, a decrease in trunk muscle mass may be associated with impaired physical function and ADL in older adults. Reduced trunk muscle mass correlates with postural alignment in older adults ^(6)^ and dysphagia in older patients with stroke ^(28)^. Furthermore, a decrease in trunk muscle mass limits the recovery of ADLs in older patients with stroke ^(25)^. Therefore, trunk muscle mass likely decreases with age in older individuals, regardless of disease status, and is associated with functional outcomes. However, most of these studies were cross-sectional or retrospective in design, making causal inferences unclear. Despite considerations such as body water content affecting BIA evaluations, the accuracy, reliability, and validity of the impedance technique are well documented. Although the number of articles included in this review was limited, BIA is a useful assessment for trunk muscle mass owing to its simplicity and non-invasiveness. Therefore, accumulating data from large-scale clinical studies in the future is crucial. Additionally, longitudinal investigations are necessary to clarify the relationship between changes in trunk muscle mass evaluated by BIA and clinical outcomes such as physical function.

### Study limitations

In this study, a scoping review was conducted to comprehensively explore and analyze existing knowledge of trunk muscle mass evaluated by BIA in older adults. However, this review had some limitations. First, the search was limited to papers written in English. As papers written in languages other than English were not included, caution must be exercised in generalizing the contents of this scoping review. Second, no qualitative evaluation of the included papers was conducted. Although qualitative evaluation is not required for scoping reviews, incorporating it could enhance the contribution of the review to evidence construction. Third, all studies included in this scoping review were published in Japan, necessitating caution in interpreting the results. To reach a consensus on evaluating trunk muscle mass using BIA, a large database incorporating age, sex, race, and other factors from diverse regions outside Japan is needed to examine and confirm the evidence. Fourth, we defined older adults as those aged ≥65 years, in line with the WHO report. However, using a criterion of ≥60 years of age would expand the scope of the survey and could change the results. Therefore, it is necessary to consider the target age in future surveys.

### Conclusions

Evaluation of trunk muscle mass using BIA is an alternative to conventional methods such as CT and MRI. However, the exact consensus on the assessment of trunk muscle mass using BIA is unclear. Furthermore, given that the data have been reported from only a limited number of countries, future research should aim to include more regions and ethnicities.

## Article Information

### Conflicts of Interest

None

### Author Contributions

Data curation, formal analysis, investigation, methodology, project administration, resources, supervision, and writing - original draft: Ryo Shiraishi. Resources, writing, review, and editing: Shunji Araki. Resources, writing, review, and editing: Masanobu Yokochi. Writing, review and editing, supervision, project administration: Masatoshi Nakamura.

### Data Availability Statement

The data supporting the findings in this study are available upon request from the corresponding author. The data are not publicly available owing to privacy or ethical restrictions.
